# Patient Perceptions of a Preventive Effect of Long-Term Botulinum Neurotoxin Therapy in Cervical Dystonia

**DOI:** 10.3390/toxins18040184

**Published:** 2026-04-12

**Authors:** Harald Hefter, Sara Samadzadeh

**Affiliations:** 1 Department of Neurology, University of Düsseldorf, Moorenstrasse 5, D-40225 Düsseldorf, Germany; harald.hefter@med.uni-duesseldorf.de; 2 Charité–Universitätsmedizin Berlin, Corporate Member of Freie Universität Berlin and Humboldt-Unverstät zu Berlin, Experimental and Clinical Research Center, D-13125 Berlin, Germany

**Keywords:** cervical dystonia, preventive effect, botulinum neurotoxin, patient perception, illness perception, patient-reported outcomes, disease progression, long-term therapy

## Abstract

Patients with cervical dystonia (CD) often believe that disease severity would have progressed beyond the pre-treatment level if botulinum neurotoxin (BoNT) therapy had not been initiated. The aim of the present study was to assess the perceptions of long-term BoNT-treated patients with CD regarding the expected course of disease severity over the next 10 years under the hypothetical assumption that BoNT therapy is discontinued. Fifty patients with idiopathic CD receiving long-term BoNT therapy were screened, and 43 patients were included. Disease severity at the day of recruitment was assessed as a percentage of CD severity at the onset of BoNT therapy (PAS-%). Patients also generated, in a standardized manner, a graph illustrating the development of CD severity since initiation of BoNT therapy. Subsequently, patients estimated the expected severity of CD after 10 years, expressed as a percentage of severity at BoNT therapy onset (PAS-STD), under the hypothetical assumption that BoNT therapy was discontinued at the time of recruitment. They additionally drew a graph depicting the anticipated progression of CD severity over the subsequent 10 years under this assumption. Furthermore, 33 of these 43 patients had previously assessed the expected development of CD severity under the assumption that no BoNT therapy had ever been performed (PAS-NO%) in an earlier study. Mean PAS-STD was significantly higher than mean PAS-% (*p* < 0.001). Comparison of mean PAS-STD with mean PAS-NO% in the 33 patients who participated in both studies demonstrated that mean PAS-STD was significantly lower than mean PAS-NO% (*p* < 0.001). Long-term BoNT-treated patients with CD believe that disease severity would worsen again if BoNT therapy were discontinued. However, they do not expect CD severity to deteriorate to the level that they believe would have been reached if no BoNT therapy had been administered. We interpret this finding as suggesting that patients perceive a preventive effect of long-term BoNT therapy.

## 1. Introduction

Cervical dystonia (CD) is the most common form of focal dystonia, with reported prevalence estimates ranging from approximately 5 to 30 cases per 100,000 individuals, depending on study design and population characteristics [1]. It typically presents in midlife and is associated with a substantial clinical burden, including abnormal head postures, involuntary muscle contractions, pain, and reduced quality of life. In addition to motor manifestations, non-motor features such as anxiety, depression, and social impairment further contribute to disease burden [2].

Intramuscular injections of botulinum neurotoxin type A (BoNT/A) or type B (BoNT/B) effectively reduce involuntary muscle contractions and pain in patients with idiopathic CD and are associated with few side effects [3,4]. Consequently, BoNT injections have become the treatment of choice for CD and have received level A recommendations from both the Royal College of Physicians (RCP) [5] and the American Academy of Neurology (AAN) [6].

BoNT injections are typically administered into overactive neck muscles, such as the sternocleidomastoid, splenius capitis, trapezius, and levator scapulae, depending on the individual pattern of dystonia. Chronic muscle overactivity may lead to structural alterations, including fibrosis and changes in muscle architecture, which can complicate accurate targeting [7]. In recent years, ultrasound-guided injection techniques have increasingly been adopted to improve precision and safety, enhancing treatment efficacy while reducing the risk of neurovascular injury and unintended toxin diffusion [8,9].

When BoNT injections are administered repeatedly before the effect of the preceding injection has completely declined, a staircase-like improvement can be observed from injection to injection. Using this treatment strategy, an improvement of approximately 50% or more can be achieved in most patients [10]. This level of improvement can be maintained over decades of treatment, provided that injections are administered regularly at three-month intervals and that no neutralizing antibodies (NABs) are induced [11,12].

Once long-term BoNT therapy has been initiated, the natural course of CD can no longer be directly observed. As more than 80% of patients with CD currently receive BoNT therapy [4], patients who remain untreated yet are followed up regularly have become rare. Consequently, little is known about the natural course of CD severity (course of disease, CoD) in the absence of BoNT therapy. Discontinuation of effective ongoing BoNT treatment solely to study disease progression without therapy would be unethical.

A patient-based method was therefore employed, involving the drawing of the course of disease (CoD-graph drawing) to explore patients’ expectations regarding disease progression in the absence of BoNT treatment [11,12]. When patients with CD were asked to speculate on the level of disease severity that would have developed without BoNT therapy, expressed as a percentage of severity at the onset of BoNT treatment, none anticipated spontaneous improvement [11]. The majority of patients (>60%) expected further worsening of approximately 50% or more in the absence of BoNT therapy [11].

These observations highlight that patients form distinct expectations about disease progression, which may extend beyond their direct clinical experience. Patient-reported outcomes (PROs) and illness perception frameworks are increasingly recognized as important determinants of treatment adherence and perceived efficacy in chronic neurological disorders. Patients’ expectations regarding disease progression are shaped not only by clinical experience but also by cognitive and psychological factors, including expectancy effects. Therefore, assessing patients’ perceptions of disease trajectories may provide complementary insights beyond objective clinical measures [13,14,15,16,17].

Thus, the perceived benefit of BoNT therapy in CD has two dimensions: patients experience symptomatic clinical improvement after treatment initiation, and they also believe that further disease worsening is prevented. This dual perception may explain the high level of treatment adherence observed among BoNT-treated patients with CD [18,19].

As a further step in exploring patients’ perceptions of BoNT efficacy, it is of interest to analyze what patients expect to happen if BoNT therapy is discontinued after a prolonged period of effective treatment. Accordingly, the present study was designed to analyze patients’ drawings of the anticipated course of CD severity over the subsequent 10 years (CoD-ST-graph), under the hypothetical assumption that BoNT therapy was discontinued at the time of recruitment. This anticipated course of disease after cessation of BoNT therapy was compared with the anticipated disease course under the assumption that no BoNT therapy had ever been administered, as assessed in a previous study conducted approximately two years earlier [11,12].

Given that patients were informed that CD is a chronic disease of the central nervous system (CNS) and that intramuscular BoNT injections represent a symptomatic treatment, it was hypothesized that patients would anticipate a degree of worsening after discontinuation of long-term BoNT therapy comparable to that expected under the assumption that no BoNT therapy had been administered. However, it is important to note that these expectations reflect subjective patient perceptions rather than objective measures of disease progression.

## 2. Results

### 2.1. Long-Term Outcome After BoNT Therapy

For the present study, 43 patients with idiopathic cervical dystonia (21 females, 22 males) were recruited. The mean age was 70.0 years (SD: 13.0; range: 38.8–87.8 years). The mean duration of BoNT treatment was 16.6 years (SD: 8.1; range: 3.0–35.6 years), corresponding to 12–144 treatment cycles.

Three patients were treated with onabotulinumtoxin type A (onaBoNT/A; mean dose: 250 U; SD: 108 U; range: 150–400 U), 27 patients with incobotulinumtoxin type A (incoBoNT/A; mean dose: 385 U; SD: 127 U; range: 200–800 U), and 13 patients with abobotulinumtoxin type A (aboBoNT/A; mean dose: 604 U; SD: 228 U; range: 250–1000 U).

The primary outcome measure was the patient-reported assessment of remaining CD severity (PAS-%) expressed as a percentage of disease severity at the onset of BoNT therapy. Mean PAS-% was 46.9% (SD: 23.3%; range: 5–130%), corresponding to a mean improvement of 53%. When patients assessed disease severity by graph drawing, the mean remaining severity indicated on the visual analogue scale (PAS-D) was 49.0% (SD: 24.1%; range: 8–147%). PAS-% and PAS-D showed a strong correlation (r = 0.9666; *p* < 1.92 × 10^−24^; Table 1).

Disease severity at the day of recruitment was assessed by the treating physician using the TSUI score (ATSUI). Mean ATSUI was 3.64 (SD: 2.38; range: 0–8) and showed a significant correlation with PAS-% (r = 0.5117; *p* < 0.000532; Table 1).

**Figure 1:**
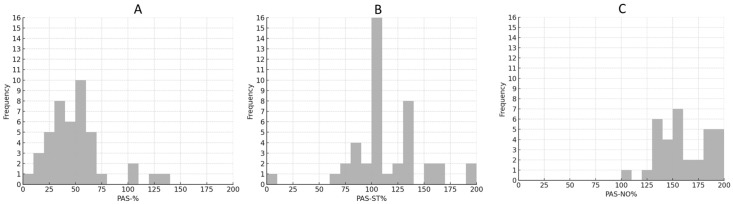
Distribution of patient-assessed cervical dystonia severity parameters: (**A**) PAS-%, (**B**) PAS-ST%, and (**C**) PAS-NO%. Histogram bars indicate the number of patients within each 5% interval. The y-axis is standardized from 0 to 16.

The distribution of PAS-% is shown in Figure 1A.

### 2.2. Suspected Outcome After Presumed Cessation of BoNT Therapy

After completing the CoD-B and CoD-A graphs, patients were asked to estimate CD severity 10 years into the future, expressed as a percentage of disease severity at BoNT therapy onset, under the hypothetical assumption that BoNT therapy would be discontinued on the day of recruitment (PAS-ST%). Patients then drew the anticipated course of disease severity over the subsequent 10 years (CoD-ST graph). The terminal point of this graph was recorded as PAS-STD.

Mean PAS-ST% was 110.0% (SD: 44.3%; range: 0–200%) and showed a strong correlation with PAS-STD (mean: 108.7%; SD: 37.5%; range: 0–164%; r = 0.893; *p* < 4.2 × 10^−15^).

The distribution of PAS-ST% is presented in Figure 1B.

### 2.3. Suspected Outcome Without BoNT Therapy

Among the 41 patients who produced continuous CoD-B, CoD-A, and CoD-ST graphs, 33 had participated in a previous study conducted approximately two years earlier [11,12], in which they had assessed the course of CD under the assumption that no BoNT therapy had been administered (CoD-NO graph). The endpoint of this graph was used as a VAS-based assessment parameter (PAS-NOD).

Prior to drawing the CoD-NO graph, patients estimated disease severity in the absence of BoNT therapy as a percentage of severity at BoNT therapy onset (PAS-NO%). Mean PAS-NO% was 156.0% (SD: 22.2%; range: 100–200%) and was strongly correlated with PAS-NOD (mean: 164.0%; SD: 32.3%; range: 100–215%; r = 0.8904; *p* < 4.0 × 10^−12^).

The distribution of PAS-NO% is shown in Figure 1C.

### 2.4. Comparison of Suspected Outcomes

Contrary to the hypothesis formulated in the Introduction, PAS-NO% was significantly higher than PAS-ST% (*p* < 1.0 × 10^−9^). There was no correlation between the experienced severity after BoNT therapy (PAS-%) and the suspected disease severity under either the assumption of no BoNT therapy (PAS-NO%) or discontinuation of BoNT therapy (PAS-ST%).

Cross-correlations between all six patient-derived parameters (PAS-%, PAS-D, PAS-ST%, PAS-STD, PAS-NO%, PAS-NOD) and the physician-derived ATSUI score are summarized in Table 1. The upper triangle displays correlation coefficients (r), and the lower triangle presents corresponding *p*-values.

**Figure 2:**
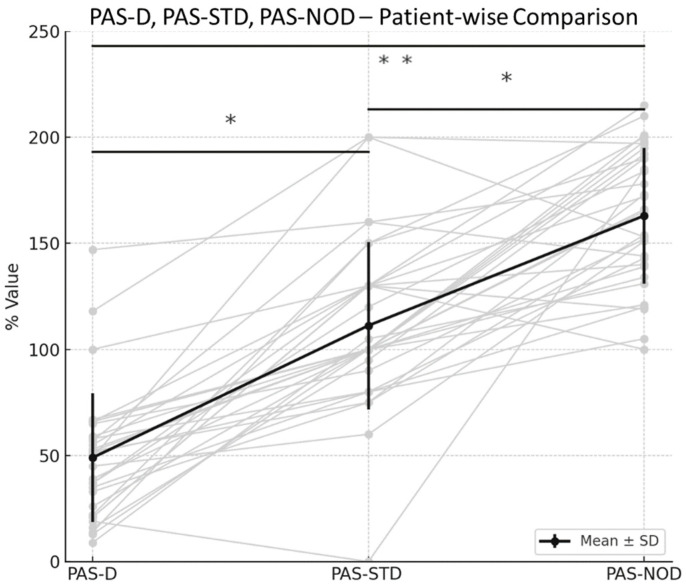
Individual data of PAS-D (left side), PAS-STD (in the middle) and PAS-NOD (right side). For each patient, lines connect PAS-D and PAS-STD, PAS-STD and PAS-NOD, and PAS-D and PAS-NOD. PAS-D was significantly lower than PAS-STD (*p* < 1 × 10^−9^), PAS-NOD was significantly higher than PAS-STD (*p* < 7 × 10^−7^), and PAS-NOD was significantly higher than PAS-D (*p* < 1 × 10^−15^). Grey lines represent individual patient trajectories, while the black line indicates the mean ± standard deviation; An asterisk (*) indicates a significant difference between mean values.

When individual PAS-D values were compared with PAS-STD values (Figure 2, left panel), only two patients did not expect an increase in disease severity following cessation of BoNT therapy. Mean PAS-STD was significantly higher than PAS-D (*p* < 1.0 × 10^−9^). These two exceptional patients are clearly identifiable in Figure 2.

When individual PAS-NOD values were compared with PAS-STD values (Figure 2, right panel), all patients expected disease severity without BoNT therapy to exceed that expected after BoNT cessation. PAS-NOD was significantly higher than PAS-STD (*p* < 7.0 × 10^−7^).

## 3. Discussion

### 3.1. Long-Term Outcome

In the present cohort of 43 long-term BoNT-treated patients with CD, male patients were slightly overrepresented (female/male ratio: 21/22 = 0.95). In other CD cohorts, the female-to-male ratio has varied widely, ranging from 0.31:1 [20] to 4.5:1 [21], with an overall mean ratio of approximately 1.7:1 [22].

The long-term outcome as assessed by the treating physician using the TSUI score was better than that reported in most previous studies. In a cohort of 221 patients with CD treated for more than 10 years, a mean TSUI score of 4.83 was reported [23], whereas the mean TSUI score in the present cohort was lower. This favorable outcome is likely related to the relatively high doses of incobotulinumtoxinA used in this study, which may be justified by its low antigenicity [24].

Patients in the present cohort were accustomed to regular BoNT injections at three-month intervals and therefore to a stable plateau of clinical improvement [10], which on average exceeded 50%. Four patients assessed their disease severity as being comparable to that at the onset of BoNT therapy. One of these patients was a primary non-responder who never experienced an improvement greater than 20%. The remaining three patients were partial secondary non-responders who had previously shown better responses. One of these patients developed complete secondary non-response due to neutralizing antibodies (NABs) against abobotulinumtoxinA but responded well after switching to incobotulinumtoxinA. In the other two patients, disease progression was observed but could be partially compensated by increasing the BoNT/A dose. This clinical constellation has previously been described as a “pseudo” secondary non-response [25,26].

### 3.2. Suspected Worsening After Cessation of BoNT Therapy

With one exception, all patients anticipated a worsening of CD severity if BoNT therapy were discontinued. The exceptional patient was convinced that improvement could be achieved through physiotherapy and mental training (Figure 2). Although patients were on a stable plateau of improvement, most experienced mild symptom worsening during the final two to three weeks of each injection cycle. It therefore appears plausible that patients extrapolated this experience to a more pronounced worsening under the hypothetical assumption that BoNT therapy was stopped on the day of recruitment.

### 3.3. Comparison of Suspected Outcomes After BoNT Cessation and Without BoNT Therapy

In all patients for whom both assumptions could be compared, the suspected worsening after cessation of long-term BoNT therapy (PAS-ST%) was significantly lower than the suspected worsening under the assumption that no BoNT therapy had ever been performed (PAS-NO%). This finding contrasts with the hypothesis formulated in the Introduction.

During treatment, all patients had been informed that CD is a chronic disorder of the CNS and that BoNT therapy represents a symptomatic rather than a causal treatment. Patients were also informed that intramuscularly injected BoNT does not penetrate the CNS [4]. Nevertheless, patients appeared to believe that discontinuation of BoNT therapy would not lead to disease worsening substantially beyond the severity experienced at the onset of BoNT treatment. This unexpected finding was not anticipated at the time the study was designed.

The absence of a correlation between experienced disease severity (PAS-%) and anticipated disease severity under both hypothetical scenarios (PAS-ST% and PAS-NO%) suggests that patients’ expectations are not solely determined by their current clinical status. Rather, these expectations may reflect broader cognitive frameworks, including illness perception and expectancy effects [27,28]. Similar phenomena have been described in other chronic neurological disorders, in which patients’ beliefs about disease progression and treatment efficacy influence subjective outcome measures, coping behavior, and treatment adherence [29,30,31]. In this context, the perceived “preventive effect” of long-term BoNT therapy observed in the present study should be interpreted as a patient-centered construct that may contribute to sustained adherence and long-term treatment engagement, rather than as evidence of biological disease modification.

Because it is unethical to discontinue effective BoNT therapy in well-responding patients solely for research purposes, controlled studies assessing disease severity after cessation of long-term BoNT therapy cannot be performed. Consequently, long-term outcomes could only be explored in patients who discontinue BoNT therapy for reasons other than secondary non-response. However, such patients are rare and are often no longer followed regularly in specialized movement disorder centers after treatment cessation.

### 3.4. Possible Explanations for Patient Perceptions

Most long-term BoNT-treated patients had experienced delays in their routine injections of up to 30 days. During the COVID-19 pandemic, the BoNT outpatient clinic at the University of Düsseldorf was completely closed for 30 days [32]. Analysis of this period revealed a worsening of CD severity of approximately 1% per day of injection delay [32]. Extrapolation of this observation suggests that disease severity could return to the level observed at BoNT therapy onset within less than one year. This contrasts with the relatively low PAS-ST% and PAS-STD values reported by patients for a 10-year period without BoNT therapy and explains this discrepancy.

In principle, it cannot be excluded that patients’ perceptions of a preventive effect of BoNT therapy may be partially correct. BoNT injections reduce sensory feedback to the brain and may influence CNS plasticity [33,34], potentially contributing to long-term changes in motor control. However, similar long-term improvements have been observed only in patients who do not develop NABs [23], and these theoretical considerations conflict with clinical observations showing disease progression over time despite effective symptom control by BoNT therapy [35].

To date, no convincing evidence indicates that CD improves with age. While some symptoms may be better tolerated in older patients [36], and improvements may occur after retirement or cessation of stressful professional activities, these factors do not support a general age-related improvement of CD severity.

## 4. Conclusions

Long-term BoNT-treated patients with cervical dystonia believe that discontinuation of BoNT therapy would result in secondary worsening of disease severity up to approximately the level observed at treatment onset. At the same time, they believe that disease severity would have been significantly greater had BoNT therapy never been initiated.

These findings suggest that patients perceive long-term BoNT therapy not only as symptomatic treatment but also as having a preventive effect on disease progression. However, this interpretation reflects subjective patient perceptions rather than objective evidence of disease modification. Future studies should aim to validate these findings in larger, multicenter cohorts and to explore longitudinal changes in patient perceptions in relation to objective clinical outcomes.

### Strengths and Limitations

A major strength of the present study is that it provides evidence that long-term BoNT-treated patients with CD perceive BoNT therapy as having a preventive effect on disease progression. Limitations include the relatively small sample size, although statistically significant differences were detected, and the inherent inability to confirm these perceptions through controlled studies assessing disease progression after BoNT therapy cessation. Additional limitations include the reliance on hypothetical scenarios, which may not accurately reflect real-world disease progression after treatment discontinuation. Furthermore, patient estimates of disease severity at onset are subject to recall bias. In addition, no standardized cognitive or psychological screening tools were used, which may limit the ability to fully exclude subtle cognitive or affective biases influencing patient responses. Finally, the cross-sectional design precludes longitudinal validation of the anticipated disease trajectories described by patients.

## 5. Materials and Methods

### 5.1. Patients and BoNT Injection at the Day of Recruitment

Between 20 January 2025 and 27 May 2025, 50 patients with CD were screened. Inclusion criteria were: (i) a diagnosis of idiopathic cervical dystonia; (ii) regular treatment with BoNT for more than 10 treatment cycles; and (iii) no interruption of BoNT therapy for more than one treatment cycle (corresponding to 3 months).

Exclusion criteria were: (i) clinically relevant disturbances of memory or mood; and (ii) BoNT treatment for disorders other than CD (e.g., hemifacial spasm or blepharospasm) at doses exceeding 10% of the dose used for CD. Exclusion of clinically relevant cognitive or mood disturbances was based on clinical assessment during routine neurological evaluation. No formal standardized cognitive or psychological screening instruments were applied.

Ultimately, 43 patients were included in the study. Two patients were unable to produce continuous graphs due to hand tremor. The BoNT preparation and dose administered on the day of recruitment (actual dose applied, ADOSE), as well as the TSUI score assessed by the treating physician on the day of recruitment (ATSUI) [10], were documented. Date of birth, sex, onset of symptoms, and onset of BoNT therapy were extracted from medical records. Duration of treatment (DURT) was calculated based on these data.

The choice of BoNT preparation and dosing was based on routine clinical practice and determined by the treating physician, in accordance with established clinical experience and published consensus recommendations. The following BoNT/A preparations were used: onabotulinumtoxinA (Botox^®^, AbbVie, North Chicago, IL, USA), incobotulinumtoxinA (Xeomin^®^, Merz Pharmaceuticals GmbH, Frankfurt am Main, Germany), and abobotulinumtoxinA (Dysport^®^, Ipsen, Paris, France). Selection of BoNT formulation was guided by physician experience, established treatment protocols within the center, and individual patient response and tolerability. Dosing was individualized based on the clinical presentation, including the pattern and severity of dystonia, the muscles involved, prior treatment response, and tolerability, and was adjusted over time according to clinical response and the occurrence of side effects [6,37].

### 5.2. Course-of-Disease (CoD) Graph Drawing

The CoD graph methodology represents an exploratory, patient-centered approach designed to capture subjective illness trajectories. While this method has been applied in our previous work, it has not yet undergone extensive external validation and should therefore be interpreted as a hypothesis-generating tool [11,12].

Patients were asked to draw three course-of-disease graphs (CoD graphs): (i) the CoD-B graph, representing disease severity from symptom onset to the first BoNT injection; (ii) the CoD-A graph, representing disease severity from the onset of BoNT therapy to the day of recruitment; and (iii) the CoD-ST graph, representing the suspected development of CD severity over the next 10 years under the hypothetical assumption that no further BoNT injections would be administered.

All graphs were produced in a standardized manner.

#### 5.2.1. CoD-B Graph

**Figure 3:**
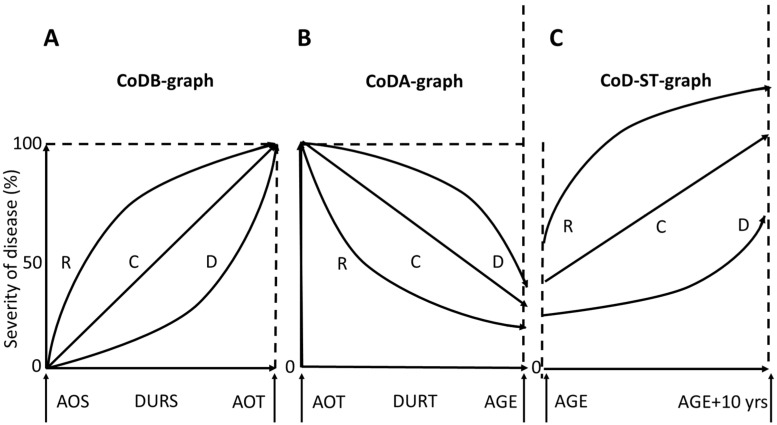
Standardized drawing of CoD-B graphs (**A**), CoD-A graphs (**B**), and CoD-ST graphs (**C**). These panels illustrate the standardized templates used for patient-drawn course-of-disease (CoD) trajectories and do not represent actual patient data. The abscissa (x-axis) of the CoD-B graph represents the time from symptom onset to the initiation of therapy (DURS; from age at onset of symptoms (AOS) to age at onset of therapy (AOT)). The abscissa of the CoD-A graph represents the duration of treatment (DURT; from age at onset of therapy (AOT) to age at recruitment (AGE)). The abscissa of the CoD-ST graph represents the subsequent 10 years following the day of recruitment into the present study. CoD graphs can be classified as R-type, D-type, or C-type based on their shape, according to previously published criteria [11,12]. R-type indicates a rapid manifestation or response pattern, D-type indicates a delayed manifestation or response pattern, and C-type indicates a continuous course.

For the CoD-B graph (Figure 3A), a square measuring 10 × 10 cm was presented. The lower left corner represented symptom onset, and the upper right corner represented CD severity at the onset of BoNT therapy (defined as 100%). Patients were instructed to draw a continuous line from the lower left to the upper right corner, reflecting disease progression before BoNT therapy (see [11,12] for details).

#### 5.2.2. CoD-A Graph

For the CoD-A graph (Figure 3B), a second 10 × 10 cm square was presented, with the right side extended by an additional 10 cm. The lower edge represented treatment duration, and the lower right corner represented the day of recruitment. Patients first estimated the remaining severity of CD on the day of recruitment as a percentage of severity at BoNT therapy onset (PAS-%). This value was marked on the extended right side of the square as a visual analogue scale (VAS) value (PAS-D). Patients then drew a continuous line from the upper left corner of the square to the PAS-D mark (see [11,12]).

#### 5.2.3. CoD-ST Graph

For the CoD-ST graph (Figure 3C), a third 10 × 10 cm square was presented with both left and right sides extended by 10 cm. The left-side mark corresponded to PAS-D. Patients then estimated the suspected severity of CD after 10 years without BoNT therapy (PAS-ST%) and marked this value on the extended right side (PAS-STD). A continuous line was drawn from PAS-D to PAS-STD to represent the anticipated disease course under this assumption.

Among the 41 patients who produced continuous CoD-B, CoD-A, and CoD-ST graphs, 33 had participated in a previous study conducted approximately two years earlier [11,12]. In that study, patients assessed the suspected severity of CD under the assumption that no BoNT therapy had ever been administered (PAS-NO%). This value was marked on the extended right side of a 10 × 10 cm square (PAS-NOD), and a continuous graph (CoD-NO graph) was drawn from the upper left corner (representing severity at BoNT therapy onset) to PAS-NOD.

For the present analysis, the following six parameters were evaluated: PAS-%, PAS-D, PAS-ST%, PAS-STD, PAS-NO%, and PAS-NOD. The shapes of the CoD graphs will be analyzed separately.

### 5.3. Study Design

Patients were informed about the purpose of the study and provided written informed consent prior to participation. After screening and inclusion, patients received their routine BoNT injection. Current CD severity (PAS-%) was then assessed, followed by completion of the CoD-B and CoD-A graphs. Patients subsequently estimated the suspected severity of CD under the assumption that BoNT therapy would be discontinued on the day of recruitment (PAS-ST%), and completed the CoD-ST graph. PAS-NO% and PAS-NOD values were extracted from medical records for patients who had participated in both the present and the previous study [11,12].

This study was designed as an exploratory, hypothesis-generating analysis. No formal sample size calculation was performed. The sample size was determined by the number of eligible patients fulfilling the inclusion criteria during the predefined recruitment period.

### 5.4. Statistical Analysis

Mean values and standard deviations were calculated for demographic and treatment-related variables. For the calculation of mean PAS-% and PAS-ST%, the two patients who were unable to draw continuous graphs due to hand tremor were included.

Non-parametric rank correlations were calculated between the six PAS parameters and ATSUI. Differences between PAS-% and PAS-ST% (as well as PAS-D and PAS-STD), and between PAS-ST% and PAS-NO% (as well as PAS-STD and PAS-NOD), were analyzed non-parametrically using the Mann-–Whitney U test.

All statistical analyses were performed using R (version 4.3.1). The dplyr package was used for data manipulation, and ggplot2 was used for data visualization.

## Figures and Tables

**Table 1:** Correlation matrix of patient-derived parameters (PAS measures) and physician-assessed TSUI score (ATSUI).

	ATSUI-II	PAS-%	PAS-D	PAS-ST%	PAS-STD	PAS-NO%	PAS-NOD
ATSUI-II	1	0.511736	0.441406	0.245087	0.207448	0.166806	0.12173
PAS-%	0.000532	1	0.965683	0.256981	0.151073	0.077129	0.058919
PAS-D	0.003865	1.92 × 10^−24^	1	0.297681	0.211698	0.094378	0.049846
PAS-ST%	0.11773	0.096202	0.058725	1	0.893045	0.178902	0.12974
PAS-STD	0.193105	0.345757	0.183939	4.21 × 10^−15^	1	0.152223	0.117282
PAS-NO%	0.35351	0.669651	0.607398	0.319174	0.152223	1	0.890398
PAS-NOD	0.499777	0.744655	0.786453	0.471763	0.117282	4.01 × 10^−12^	1

Values in the upper triangle represent correlation coefficients (r), and values in the lower triangle represent corresponding *p*-values. Abbreviations: ATSUI, Toronto Western Spasmodic Torticollis Rating Scale (Tsui score) assessed by the physician at the day of recruitment; PAS-%, patient-assessed CD severity at the day of recruitment expressed as a percentage of severity at BoNT therapy onset; PAS-D, visual analogue scale (VAS)-based patient assessment of CD severity at the day of recruitment; PAS-ST%, patient-estimated CD severity 10 years after discontinuation of BoNT therapy (percentage); PAS-STD, VAS-based estimate of CD severity 10 years after discontinuation of BoNT therapy; PAS-NO%, patient-estimated CD severity under the assumption that no BoNT therapy had ever been administered (percentage); PAS-NOD, VAS-based estimate of CD severity under the assumption that no BoNT therapy had been administered.

## Data Availability

The original contributions presented in this study are included in the article. Further inquiries can be directed to the corresponding author.

## Abbreviations

Abbreviation	Definition
aboBoNT/A	Abobotulinum neurotoxin type A
ADOSE	Actual dose applied at the day of recruitment
ATSUI	Actual TSUI score at the day of recruitment
BoNT	Botulinum neurotoxin
CD	Idiopathic cervical dystonia
CNS	Central nervous system
CoD	Course of disease
CoD-A-graph	Graph representing the course of disease severity after onset of BoNT/A therapy
CoD-B-graph	Graph representing the course of disease severity before onset of BoNT/A therapy
CoD-NO-graph	Graph representing the course of disease severity under the assumption that no BoNT therapy had been initiated
CoD-ST-graph	Graph representing the course of disease severity under the assumption that BoNT/A therapy was stopped at the day of recruitment
DURT	Duration of treatment
incoBoNT/A	Incobotulinum neurotoxin type A
mean	Mean value
NAB	Neutralizing antibodies
onaBoNT/A	Onabotulinum neurotoxin type A
PAS-D	Patient’s assessment of CD severity at the day of recruitment
PAS-%	Patient’s assessment of CD severity at the day of recruitment, expressed as a percentage of severity at BoNT therapy onset
PAS-NOD	Patient’s assessment of CD severity assuming no BoNT therapy had been initiated
PAS-NO%	Patient’s assessment of CD severity assuming no BoNT therapy had been initiated, expressed as a percentage
PAS-STD	Patient’s assessment of CD severity under the assumption that BoNT therapy was discontinued at the day of recruitment
SD	Standard deviation
TSUI	Severity score of cervical dystonia according to Tsui et al.
U	Mouse unit
VAS	Visual analogue scale
